# Towards real-time diffuse optical tomography with a handheld scanning probe

**DOI:** 10.1364/BOE.549880

**Published:** 2025-03-26

**Authors:** Robin Dale, Nicholas Ross, Scott Howard, Thomas D. O’Sullivan, Hamid Dehghani

**Affiliations:** 1 University of Birmingham, Medical Imaging Lab, School of Computer Science, University Rd W, Birmingham, B15 2TT, UK; 2 University of Notre Dame, Department of Electrical Engineering and Bioengineering Program, 275 Fitzpatrick Hall, Notre Dame, Indiana, 46556, USA

## Abstract

Diffuse optical tomography (DOT) performed using deep-learning allows high-speed reconstruction of tissue optical properties and could thereby enable image-guided scanning, e.g., to enhance clinical breast imaging. Previously published models are geometry-specific and, therefore, require extensive data generation and training for each use case, restricting the scanning protocol at the point of use. A transformer-based architecture is proposed to overcome these obstacles that encode spatially unstructured DOT measurements, enabling a single trained model to handle arbitrary scanning pathways and measurement density. The model is demonstrated with breast tissue-emulating simulated and phantom data, yielding - for 24 mm-deep absorptions (*μ*_*a*_) and reduced scattering (*μ*_*s*_′) images, respectively - average RMSEs of 0.0095±0.0023 cm^−1^ and 1.95±0.78 cm^−1^, Sørensen-Dice coefficients of 0.55±0.12 and 0.67±0.1, and anomaly contrast of 79±10% and 93.3±4.6% of the ground-truth contrast, with an effective imaging speed of 14 Hz. The average absolute *μ*_*a*_ and *μ*_*s*_′ values of homogeneous simulated examples were within 10% of the true values.

## 1. Introduction

### 1.1. Diffuse optical tomography

Diffuse Optical Tomography (DOT) is a non-invasive imaging technique used to map functional changes in human tissue up to 2-3 cm below the skin. In the context of breast imaging, DOT is a promising tool for diagnosing [1] and classifying [2,3] tumors, and for monitoring individual responses during treatment. Changes in DOT-derived functional markers such as tissue optical index (TOI) between pre- and mid- treatment scans provide a more reliable predictor of pathologic Complete Response (pCR) in neoadjuvant chemotherapy (NAC) patients, as compared to structural imaging techniques such as ultrasound, without the high-cost of magnetic resonance imaging or the potentially harmful radiation exposure associated with positron emission tomography [4].

DOT relies on the relatively low and spectrally-varying absorption profiles of common biological chromophores (oxy- & deoxy-haemoglobin (HbO & Hb), water, lipids) to near-infrared light (600–1000 nm), to measure their spatial distributions in biological tissue. In traditional Continuous-Wave (CW-) DOT, light is emitted into the tissue at a constant intensity (the incident signal), and measured at detectors positioned 1-5 cm away (the boundary signal). The high rate of photon scattering in tissue leads to an approximately isotropic diffusion of the incident light through the tissue. The boundary measurement therefore captures a small portion of the multiple-scattered photons, with the Source-Detector Separation (SDS) being proportional to the maximum depth which the measured photons have penetrated. The attenuation of the signal over time is used to infer changes in the absorption of the intervening tissue (assuming constant scattering), and combining measurements from multiple wavelengths allows estimation of changes of specific chromophores based on their known absorption spectra.

In Frequency-Domain DOT (FD-DOT) the incident signal is intensity-modulated, and both attenuation and phase shift are computed from the boundary signal. The phase measurement is indicative of mean photon time-of-flight, and provides an additional indirect signal of both absorption and scattering effects. Measuring both amplitude and phase enables recovery of absolute values of absorption ( $\mu_{a}$ ) and reduced scattering ( $\mu_{s}^{\prime }$ ) coefficients, and thus more accurate assessment of chromophore concentrations [5]. Furthermore, since scattering rate is largely determined by cell size and density, spatial variation in $\mu_{s}^{\prime }$ provides an addition indicator of increased or decreased structural homogeneity associated with certain types of tumors [6,7].

FD-DOT systems have historically been heavy, complex, and challenging to operate but recent developments in hardware design and fabrication have significantly improved usability and fidelity. For example, Stillwell et al. have developed a FD probe capable of high-speed, high-fidelity measurements, while scanning over multiple wavelengths and modulation frequencies, with a form factor similar to that of modern ultrasound systems [8,9]. Such devices open up new practical opportunities for clinical DOT, given appropriate algorithms to exploit the complex and information-rich data streams.

Bulk $\mu_{a}$ & $\mu_{s}^{\prime }$ recovery is straightforward given accurate multi-distance or multi-frequency FD-DOT measurements using either a look-up table or least-squares fitting to a physics-based ’forward’ model, however 2D or 3D image reconstruction (referred to as Topography and Tomography respectively) is generally required in clinical applications to establish the spatial characteristics and true contrast of tissue features. DOT image reconstruction requires multiple, overlapping and multi-distance measurements, and is challenging due to the ill-posed and non-unique nature of the inverse problem when the number of unknown values (optical properties) is far greater than the number of known values (measurements). The DOT inverse problem has often been solved by minimising the difference between experimental measurements and those simulated with a numerical forward model - either analytical (e.g. NIRFAST [10]) or Monte Carlo [11]. However, Deep-Learning (DL) can also be used, and presents a statistical approach to reconstruct tissue optical properties directly from boundary measurements.

### 1.2. Deep-learning diffuse optical tomography

In DL-DOT, a multi-layered neural network is trained via gradient descent on many pairs of simulated DOT measurements (inputs) and tissue volumes (outputs), to emulate the DOT inverse function [12]. In some cases the DOT inverse problem has been solved with Fully-Connected (FC) layers alone. Feng et al. [13] used a three-layer FC network to recover $\mu_{a}$ for a 2D circular phantom, and reported significantly improved image quality as compared to conventional tikhonov regularisation, without the use of spatial parameter sharing (e.g. convolutions) in either the measurement of image domain - the final layer of the network had n nodes corresponding to the N nodes in the circular mesh. Zou et al. [14] trained an autoencoder-style FC network on simulated and phantom data to solve the forward and inverse DOT problems, using structural constraints from co-registered ultrasound. The majority of published DL-DOT models adopt a FC+CNN architecture. This is a canonical approach for end-to-end image reconstruction, which was first popularised (and named ’AUTOMAP’) in [15] for MRI imaging, and has since been adapted to other common imaging modalities including PET [16] and CT [17]. It employs an initial FC network to map from signal- to image- domains, followed by a CNN for image-domain transformations, followed by a final single-filter convolution (conv) operation to integrate spatial features to produce a final image output. Yedder et al. [18] presented a FC+CNN model inspired by AUTOMAP for 2D $\mu_{a}$ imaging of breast mimetic phantoms using a statically positioned FD probe, which they subsequently developed in [19] with a learned data adaptation network, and in [20] by using spatial attention in the CNN layers. Yoo et al. [21] used the convolutional framelets theory to demonstrate how an AUTOMAP style model comprised of a single fully-connected layer followed by a 3D Convolutional Neural Network (CNN) with an encoder-decoder structure can theoretically invert the Lippman-Schwinger integral equation, providing justification for such an architecture for modeling photon propagation and solving the DOT inverse problem. They demonstrated their model by recovering 3D $\mu_{a}$ in biomimetic phantoms and animal tissue. Deng et al. [22] found that extending this architecture by adding a separately trained U-Net improved anomaly localisation and contrast. The model they proposed, ’FDU-Net’, was the first DL-DOT model trained exclusively on simulated data and demonstrated in vivo for 3D imaging of a human breast, thereby demonstrating the viability of this approach for clinical application of DL-DOT. While most published DL-DOT models have been trained on CW- DOT data to predict $\delta \mu_{a}$ or $\mu_{a}$ (assuming constant scattering), in [23], a DL network based on the FDU-net structure was demonstrated to accurately reconstruct both $\mu_{a}$ and $\mu_{s}^{\prime }$ simultaneously, directly from multi-distance FD-DOT measurements. Furthermore it was found that the proposed model offers enhanced benefits over standard DOT in the multi-parameter case, including reduced cross-talk between parameters.

The DL-DOT approach offers several benefits as compared to analytical DOT. Firstly, reconstruction is 3-4 orders of magnitude faster [20,22,23] because it does not require calculation of a forward model, and the inverse model consists primarily of parallelizable matrix multiplications. Secondly, the need for expert-turned parameters is front-loaded to the training stage, meaning that the trained model can be used without detailed knowledge of the algorithmic techniques. Thirdly, multiple studies have found DL-DOT to produce higher fidelity reconstructions than analytical methods in terms of optical property accuracy and spatial characteristics of tissue [13,14,20,22,23].

### 1.3. Towards handheld scanning

To clarify the relationship between DOT system design, imaging protocol, and reconstruction algorithm, three different imaging paradigms are considered: *Fixed-Array*, in which an array of sources and detectors is positioned statically on the tissue boundary (this is the traditional paradigm used in the vast majority of past DOT studies); *Structured Scanning*, in which a mobile probe housing a small source/detector array is used to take measurements in a predefined spatial structure (e.g. a grid); and *Unstructured Scanning*, in which a mobile probe is used to take measurements in a freehand manner, allowing arbitrary movement with a given number of degrees of freedom and within given spatial & temporal limits. Both structured and unstructured scanning techniques exploit the increased miniaturisation of DOT hardware for greater practicality and patient comfort. They enable high-density overlapping measurements - prerequisite for accurate 3D reconstruction - with a smaller, cheaper, and easy-to-use system, and without the need to compress or otherwise constrain the breast, as is generally required to ensure consistent coupling for high-density fixed-array DOT. The additional benefit of unstructured over structured scanning lies in the flexibility to determine the scanning protocol on a per-scan basis. This would allow a clinician conducting a scan to account for differences in tissue morphology and individual patient needs. When combined with a sufficiently high-speed reconstruction algorithm it could also enable a new paradigm for clinical DOT: continuous image-guided scanning, whereby a clinician could perform a fast initial ’scout’ scan whilst visualising the tissue in real time, followed by an more thorough scan to target an identified region of interest with higher resolution. In both ’scanning’ paradigms, positional information is recorded as an auxiliary to each averaged measurement, either via a video [24], inferred from the timings of the scan structure [23], or directly measured with an integrated motion tracker [8], to allow integration of spatial information by the chosen reconstruction algorithm. For breast tumor imaging, where the pathological relevant features are assumed to be stable over the course of minutes, it is standard practice to average or otherwise down-sample the DOT measurements to remove high-frequency system noise and fluctuations such as cardiac and respiratory signals, and recover a single representative measurement for each source/detector position.

From the point of view of traditional iterative imaging algorithms, the different imaging paradigms present minimally differing cases of the same optimisation problem, formulated as:

(1) $$\underset{\mu }{\text{minimize}}|f(\mu )-Y|+\lambda (\mu )$$

where μ is the vector of optical properties to be estimated, f is the forward model, Y is the measured data, and λ is a regularisation term [10]. From the DL- point of view however, the different data structures afford significantly different model designs to efficiently integrate the measurement and contextual information across the set of measurements.

In models where the first layer is FC (including all of those mentioned in 1.2), the input measurements retain no spatial information relating to the measurement protocol (e.g. channel position, SDS), since each input feature (measurement) is connected to each node in the next layer by an individual weight. This is an example of *implicit spatial encoding*, meaning that the relationship between each measurement and reconstruction voxel is inferred by the position of the measurement in the input vector, which must therefore be consistent across both training and testing data in order for the network to learn effectively. This approach has the benefit of being applicable to any combination of probe and tissue geometries, however it generally requires that a new set of geometry-specific training data is generated for each data collection. Another cost of this flexibility is that the FC layer is inefficient in terms of training time and data requirements in cases where relevant spatial information between measurements is discarded (i.e. in structured scans and in many cases of fixed-array, since there is generally some pattern in source/detector placement).

Full-conv architectures can also be used, in which the measurements are passed as a set of images to an initial 2D conv network for feature extraction, instead of an FC layer. The 2D conv layers apply a set of shared filter weights across the input, thereby learning a generalised mapping function between measurements and optical property configurations. This is an example of *intrinsic spatial encoding*, meaning that positional information is not explicitly encoded in the input, but is automatically preserved due to the structure of the node connections. Full-conv models are more efficient in cases where the spatial relationship between the input measurements and underlying optical properties is consistent, such as structured scans, and has also been shown to improve the classification accuracy of a functional near-infrared spectroscopy-based brain-computer-interface, trained on limited fixed-probe data [25], in which the probe design meets the criteria of translational invariance. They can also be extended to handle additional degrees of freedom, such as probe rotation [26]. However, the full-conv architecture does not scale effectively to high resolution unstructured scanning. Firstly, because the measurement image size and spatial resolution are pre-defined in the training data (meaning that non-comprehensive scans must be interpolated, leading to error and inefficient training), and secondly because the input layer size scales exponentially with spatial resolution, leading to a further trade-off between training efficiency and precision. Furthermore, the translational-invariance assumption of the conv input layer, while sufficient for structured scans over flat surfaces (e.g. optical phantoms, as in [26]), has not yet been demonstrated with realistic tissue volumes, and is likely to require additional steps of post-processing to handle significantly non-flat surfaces. In summary, while both the FC-CNN and Full-conv approaches have been demonstrated to produce accurate reconstructions under certain conditions, both incur significant practical limitations as additional degrees of freedom are added to the measurement paradigm, and neither is therefore appropriate to address the unstructured scanning case.

Over the past few years, DL models based on the transformer architecture have become dominant in a wide range of learning tasks involving deep feature representation, and have been shown to outperform the CNNs and Recurrent Neural Networks (RNNs) on many benchmark tests, especially for sequence-to-sequence tasks which require long distance dependencies [27,28], such as text translation and large language models. Transformers are well suited for these tasks because they use explicit positional encoding and self-attention (explained fully in 2.3) to calculate statistical relationships between input data-points. The units of input data that transformers manipulate, called ’tokens’, can represent various types of data points (words, patches of an image, etc.) depending on the task, and generally encode information relating to both the data content and explicit positional context of that data. In medical imaging, Vision Transformers (ViTs) have been applied for a wide range of clinically relevant tasks including image segmentation, labeling, and disease classification [29]. Some recent works have investigated ViTs for DOT processing tasks. Xue et al. designed a U-Net with hierarchical ’attention-convolution’ modules (APU-Net) to enhance the quality of analytically derived DOT images [30]. Zou et al. developed a dual-input transformer model ’USDOT-Transformer’, which predicts pCR in NAC patients from co-located DOT images and ultrasound images, with higher accuracy than a multi-modality CNN-based model [31], highlighting the benefit of the the transformer’s self-attention mechanism to establish global context to improve prediction.

In this work, a transformer encoder architecture is proposed to model the DOT inverse problem, to enable real-time reconstruction of $\mu_{a}$ & $\mu_{s}^{\prime }$ directly from FD unstructured scan data. This is the first transformer model used for DOT measurement encoding, and the first DL-DOT to use explicit spatial encoding. The transformer treats the unstructured DOT measurements as a sequence of heterogeneously related data points, and extracts spatial information about the probed tissue in the same way that large language models extract the meaning from the series of words in a sentence. This technique enables a single trained network to handle arbitrary measurement sequences, making it uniquely suited to the unstructured scanning paradigm. The model is demonstrated with both a simulated dataset, and experimental FD-DOT data collected with a fiber-based mechanical probe and a tumor-emulating optical phantom.

## 2. Methods

### 2.1. Experimental data

**Fig. 1.**
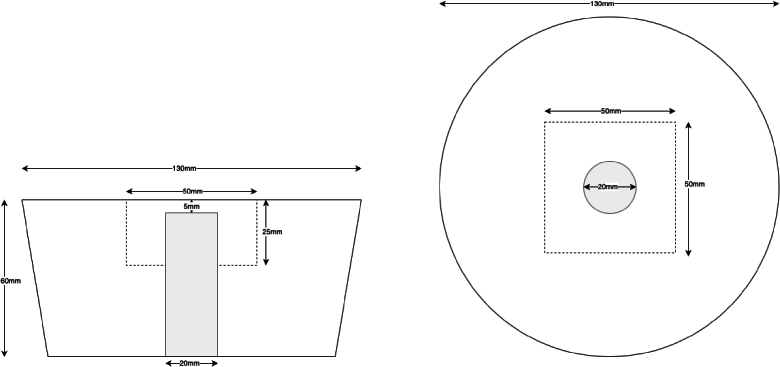
Side-view and top-view diagrams of the tumor-emulating optical phantom used in FD-DOT data collection. The gray area indicates the tumor-emulation anomaly, and the dashed line indicates the target reconstruction area.

**Fig. 2.**
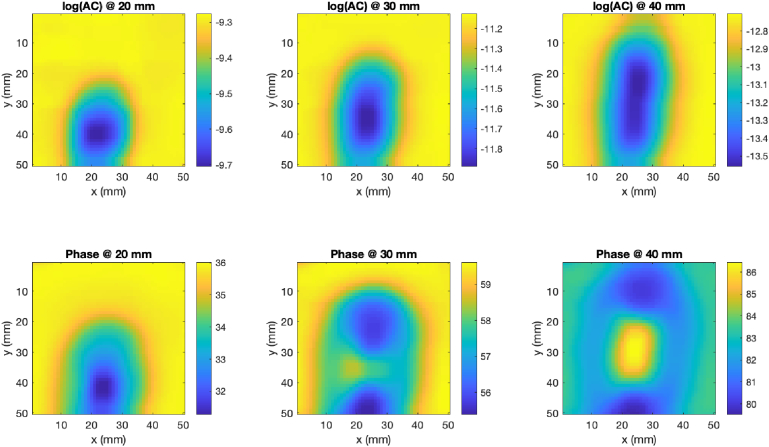
Log(amplitude) and phase (degree) data from a 1 mm resolution phantom scan with a FD-DOT system and mechanical probe @ 830 nm

Experimental data were collected by scanning an optical phantom containing a tumor emulating anomaly with a fiber based FD-DOS system (ISS Imagent 2 [32]). A single, dual-wavelength source (690 and 830 nm) was used, with three detectors positioned linearly at 20, 30, 40 mm from the source. A mechanical probe structure was built to achieve precise, high resolution scans, which were then undersampled to simulate different unstructured scanning pathways. The sources and detectors were secured by a clamp, and the phantom was attached to a motorised xy-stage, which was programmed to move in lateral x and y directions whilst remaining in contact with the probe. The phantom was a tapered cylinder shape with homogeneous background optical properties except for a single cylindrical anomaly, shown in Fig. 1. Scan data were collected in co-located horizontal and vertical grid scans over the central 50 × 50 mm region of the phantom surface, with the probe oriented perpendicularly to the direction of the scan. The construction process for the phantom is described in [23], and details of the gridscan protocols and phantom properties are shown in Table 1. An example of the experimental data is shown in Fig. 2.

**Table 1.** Parameters of simulated and experimental FD-DOT data

		Simulated scans	Experimental scans	
Scan resolution (mm)		1	1	
Modulation frequency (MHz)		100	100	
Wavelength (nm)		-	690	830
Substrate optical properties ( $mm^{-1}$ )	$\mu_{a}$	0.005 ± 0.002	0.0047	0.0024
	$\mu_{s}^{\prime }$	0.98 ± 0.2	1.36	1.05
Contrast (anomaly/substrate)	$\mu_{a}$	1.5 - 3.5	2.21	2.04
	$\mu_{s}^{\prime }$	1.5 - 2.5	1.61	1.76
Number of anomalies		0-5	1	
anomaly radius (mm)		5-15	10	
anomaly shape		sphere, cylinder, cuboid	cylinder	
anomaly minimum depth (mm)		0 - 20	5	

### 2.2. Simulated data

60,000 unstructured FD-DOT scans were simulated with NIRFAST [10], using a finite element model with 589,663 tetrahedral elements (1.04 ± 0.28 ${\mathrm{mm}}^{3}$ ), with dimensions matching those of the phantom described in 2.1, with sources and detectors placed so as to emulate the experimental probe geometry (20, 30, 40 mm SDS). For each example, the parameters of the phantom model were independently sampled from the distributions shown in Table 1. The optical property distributions were based on population level studies of human breast and tumor tissue [7,33,34]. Furthermore, for each example, a random selection of 256 probe positions was selected, and all other sources and detectors were deactivated. Data were simulated for each detector at each active probe position, and noise was added to the complex FD measurements according to an empirically-derived noise model based on multi-distance, multi-frequency phantom measurements [35]. Noise was modeled as a complex gaussian, with equal variance in the real and imaginary parts. The variance was determined from an empirically derived two-segment, piecewise log-linear fit that provides a constant noise floor below −51dBm and an amplitude-dependent linear increase in noise (in dB) for signals above that threshold. Because the measured amplitude is frequency-dependent, the noise variance is inherently frequency-dependent as well. Amplitude and phase components were taken from the noise-added measurements.

The optical properties of the central scan area ( 50×50×24 mm) of each tissue model were interpolated onto a regular grid of voxels with 2 mm resolution, to create a reconstruction target with dimensions 25×25×12×2 corresponding to $\left(x\times y\times z\times \mu_{a}/\mu_{s}^{\prime }\right)$ . The simulated data were split into training (50,000 examples), validation (5,000) and test (5,000) sets. Each of the validation and test examples was randomly under-sampled between 1-256 measurement positions to emulate varying scan durations and pathways.

### 2.3. Neural network architecture

**Fig. 3.**
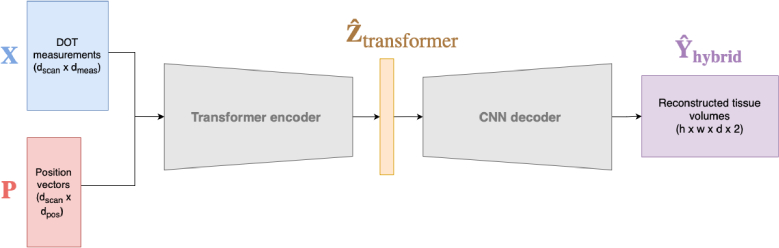
Diagram of the hybrid DL-DOT model. The trained transformer encoder and CNN decoder are combined to directly predict tissue parameters from unstructured measurements.

A neural network was trained to predict absolute values of $\mu_{a}$ and $\mu_{s}^{\prime }$ in 3D directly from a sequence of unstructured DOT measurements. The hybrid architecture, illustrated in Fig. 3, consists of a transformer encoder which maps a set of unstructured DOT measurements from a single scan to a ’latent’ volume - a learned, low-dimensionality representation of a tissue volume which has many feature channels but low spatial resolution - followed by a 3D CNN decoder.

#### 2.3.1. Inputs

**Fig. 4.**
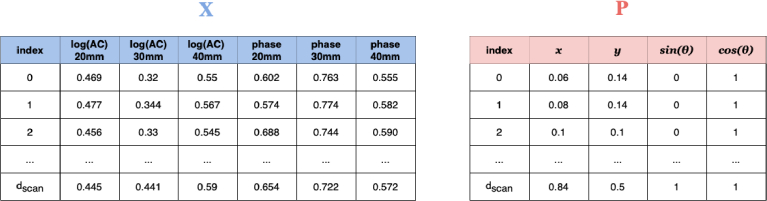
Example of unstructured FD-DOT scan data (X) , and the corresponding context data (P) describing the lateral position and rotation of the probe (both normalised).

The inputs to the transformer encoder for each DOT scan (illustrated in Fig. 4) are a measurement matrix $X\in R_{d_{scan}\times d_{meas}}$ , where $d_{scan}$ is the scan length (i.e. number of discrete probe positions), and $d_{meas}$ is the number of measurements corresponding to each probe position, and a corresponding context matrix $P\in R_{d_{scan}\times d_{pos}}$ , in which econsow represents the positional information corresponding to each row in X , and $d_{pos}$ is the number of context features. In this case $P_{i}=[x_{i},y_{i},cos(\theta_{i}),sin(\theta_{i})]$ , where $x_{i},y_{i}$ are the euclidean coordinates of the probe and $\theta_{i}$ is the rotation of the probe corresponding to measurement i , with respect to a set origin point and orientation.

#### 2.3.2. Input embedding

**Fig. 5.**
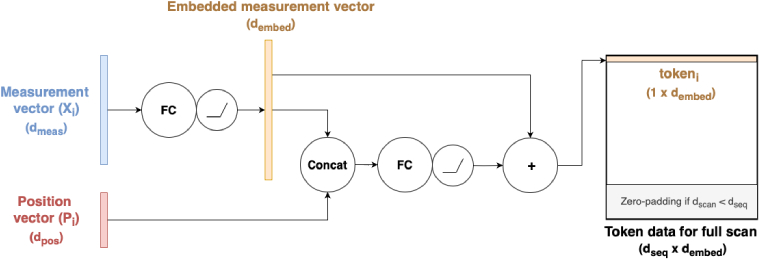
Diagram of input embedding layer applied for a single measurement and the corresponding position vector.

The input embedding layer (illustrated in Fig. 5) independently encodes each measurement vector $X_{i}$ and its corresponding position vector $P_{i}$ into a single input ’token’ through a series of learned transformations. Firstly, the measurement vector is passed to a FC layer with $d_{embed}$ nodes, the output of which is concatenated with that measurement’s corresponding position vector, and passed to another FC layer with $d_{embed}$ nodes. The positional embedding is added to the embedded measurement producing a $1\times d_{embed}$ vector (the token) for each measurement, which represents the measurement’s combined feature and positional information. The encoded measurements for a single scan are passed to subsequent layers as a $d_{seq}\times d_{embed}$ matrix, where $d_{seq}$ is the maximum sequence length that the transformer is trained to handle. In cases where the scan $d_{scan}<d_{seq}$ , the token matrix is padded with zeros to achieve the required shape, thereby enabling the network to process multiple scans of varying lengths in parallel.

#### 2.3.3. Token-to-token transformer layers

The transformer layers integrate information between embedded measurements using a multi-headed self-attention mechanism, which allows each token to search for a specific combination of features based on its own current state and its relationship to the states of the other tokens. The attention mechanism in each layer utilises three learned linear projections of the input measurements, commonly denoted as Key, Query, and Value matrices. Query ( $Q\in R_{d_{seq}\times d_{embed}}$ ) represents the features that each token is ’searching’ for amongst the other tokens. Key ( $K\in R_{d_{seq}\times d_{embed}}$ ) represents the existing values of the same searchable features for each token, and Value ( $V\in R_{d_{seq}\times d_{embed}}$ ) represents the feature information which is passed on to the next transformer layer, not necessarily the same features used for attention calculation. The attention weights are calculated as:

(2) $$A=softmax(QK^{T})$$

Where $A\in R_{d_{seq}\times d_{seq}}$ is the attention weight matrix, Q and K are the Query and Key matrices, and the row-wise softmax function ensures that the attention weights for each token sum to 1. The transformer layer’s output tokens are then calculated by:

(3) $$X_{i+1}=V^{T}A$$

In multi-headed attention, multiple instances of K,Q,V, and A are computed, each having independent weights, allowing different heads to specialise for different features. The outputs of the multiple heads are concatenated to create the layer’s output. The number of features in each attention head is generally chosen to be the embedding dimension divided by the number of heads, in order to maintain the embedding dimension throughout multiple transformer layers. After the final transformer layer, the output tokens are averaged into a single global token, which is passed to a final fully-connected layer with $d_{latent}$ nodes.

#### 2.3.4. CNN decoder

The decoder is composed of a series of 3D conv and up-sampling layers, which integrate spatially local features in a hierarchical manner, to up-sample the latent volume to the original target resolution.

### 2.4. Neural network training

**Fig. 6.**
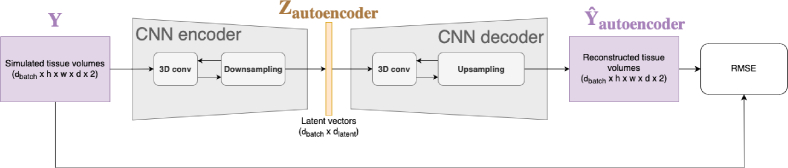
Diagram of training step 1. The CNN autoencoder is trained (self-supervised) to encode and decode tissue volumes, passing a ’latent’ representation at the architectural bottleneck.

The two modules (the transformer encoder and CNN decoder) were trained separately, with the decoder trained first as part of a self-supervised autoencoder (Fig. 6). This training pipeline significantly reduces the complexity of the encoder learning task, as compared to training in an end-to-end manner, and also allows the decoder to be validated independently before training the encoder.

The autoencoder was trained on the ground-truth tissue model optical properties in a self-supervised manner, meaning that the input data and the target labels were identical for each training example (Fig. 6). The autoencoder has a CNN encoder module, (not used in the final DOT model) composed of sequential 3D conv and max-pooling layers which are symmetrical to the up-sampling in the decoder module, such that the encoder’s input has the same dimensionality as the decoder’s output, and the centre of the network is an informational bottleneck, where a single vector $Z\in R_{d_{latent}}$ is passed from encoder to decoder. When trained on identical inputs and outputs, the encoder and decoder function as dimensionality reduction and reconstruction functions, respectively, which specifically preserve the most relevant features for minimising the given loss function. Once the autoencoder was fully-trained, all training and validation examples were encoded and resulting latent vectors were saved.

**Fig. 7.**
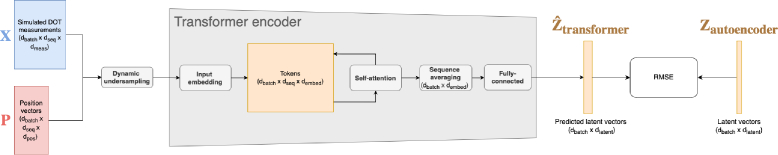
Diagram of training step 2. A transformer encoder is trained to predict latent vectors from the corresponding unstructured DOT measurements.

The transformer encoder was then trained to predict the latent volumes from the corresponding unstructured measurements (Fig. 7). During training, the input data batches were dynamically under-sampled to emulate varying scans, by generating randomised binary masks $M\in R_{d_{batch}\times d_{seq}}$ , which was applied to the inputs to each transformer layer to prevent the model accessing information from the masked tokens. This technique reduces over-fitting to the training data (because a different subset of each training example’s measurements are sampled each time it is batched), and also enables the trained model to handle scans of varying sparsity with a single set of weights. After training the transformer encoder and the CNN decoder were combined sequentially to produce the final hybrid model which maps directly from unstructured measurements to optical properties, Fig. 3.

### 2.5. Evaluation

The trained transformer+CNN model was used to reconstruct 3D $\mu_{a}$ and $\mu_{s}^{\prime }$ images for all the simulated and experimental test examples. The reconstructions were evaluated independently via three quantitative metrics: Root-Mean-Squared-Error (RMSE, Eq. (4)) indicating the overall accuracy of the absolute optical property values, Sørensen–Dice Coefficient (SDC Eq. (5)), indicating the spatial similarity of the reconstructed anomalies, and Contrast Ratio (CR, Eq. (6)) indicating the ratio between the reconstructed anomaly/background contrast and the ground truth.

(4) $$RMSE(\hat{y},y)=\sqrt{\frac{1}{N}\sum_{i=1}^{N}{\left({\hat{y}}_{i}-y_{i}\right)}^{2}}$$

(5) $$SDC(\hat{y},y)=\frac{2|anom(\hat{y})\cap anom(y)|}{|anom(\hat{y})|+|anom(y)|}$$

(6) $$CR(\hat{y},y)=\frac{\left\langle {\hat{y}}_{anom(\hat{y})}\right\rangle }{\left\langle {\hat{y}}_{bg(\hat{y})}\right\rangle }/\frac{\left\langle y_{anom(y)}\right\rangle }{\left\langle y_{bg(y)}\right\rangle }$$

Where $y_{i}$ and ${\hat{y}}_{i}$ are the ground-truth and reconstructed optical property values for either $\mu_{a}$ or $\mu_{s}^{\prime }$ at voxel i , and anom(x) and bg(x) are functions that return the set of indices corresponding to anomaly and background voxels of x , respectively, defined by the condition that the Min-Max normalized values of x are greater than 0.5 for the anomaly voxels and less than 0.5 for the background voxels.

To evaluate the transformer model for anomaly characterisation, all metric scores were computed for each wavelength for each of 2,000 simulated test examples in which an anomaly was present in either $\mu_{a}$ and $\mu_{s}^{\prime }$ . Each phantom scan was under-sampled 2,000 times to emulate multiple scans of the same volume. To evaluate bulk optical property recovery with a short scan, RMSE was calculated for the simulated examples with 0 anomalies (i.e. simulating homogeneous tisse) and < 5 measurements,

## 3. Results

**Fig. 8.**
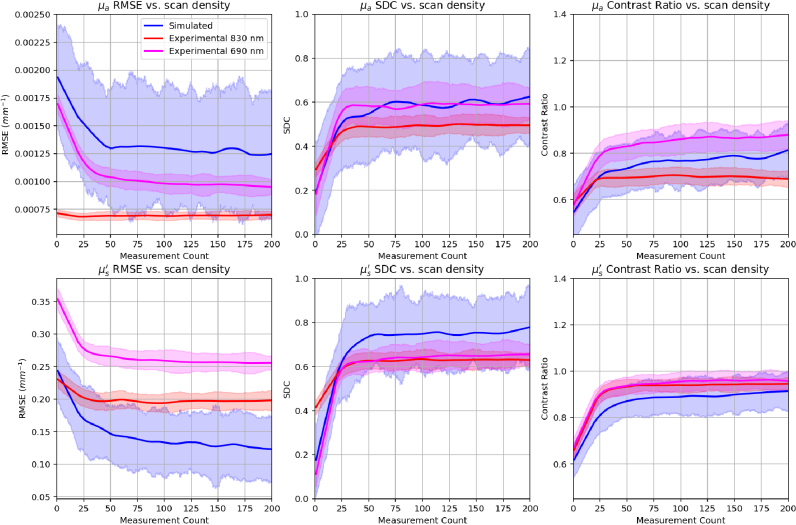
LOESS curves fitted to the Root-Mean-Squared-Error (RMSE), Sorensen-Dice coefficient (SDC) and Contrast Ratio scores for simulated and experimental DOT reconstructions as a function of measurement density.

The metric scores for the anomaly test examples are shown in Fig. 8 by LOESS curves fitted to the scores as a function of scan density. The curves are regressed within a ±20 sliding window to capture local trends as more measurements are added to the scan. The curves are shown ranging from 1-200 measurements per example (i.e. from a single measurement to a dense scan of the 50x50 mm scan region) for visibility, and because all scores were almost constant between 200-256 measurements. For both wavelengths, the curves reveal a general trend of lower RMSE, higher SDC and higher CR for scans with using more measurements. The RMSE scores of the simulated examples with homogeneous optical properties and with <=5 measurements are shown in Table 2. The metric scores corresponding to test examples with 195-200 measurements are shown in Table 3. The parameters of each dataset, including optical property distributions are shown in Table 1. Please note the RMSE values for $\mu_{a}$ and $\mu_{s}^{\prime }$ are given in $\left(m^{-1}\right)$ and $\left(cm^{-1}\right)$ respectively for conciseness.

**Table 2.** RMSE scores for 1-5 measurement scans of simulated tissue volumes without tumor-emulating anomalies

	$\mu_{a}$		$\mu_{s}^{\prime }$	
	RMSE $\left(m^{-1}\right)$	RMSE as % of true value	RMSE $\left(cm^{-1}\right)$	RMSE as % of true value
Simulated	0.471±0.355	8.33±6.31	0.468±0.232	5.1±2.37

**Table 3.** Metric scores for high-density (190-200 measurements) scans of the phantom, and simulated tissue models with tumor-emulating anomalies

	$\mu_{a}$			$\mu_{s}^{\prime }$		
	RMSE $\left(m^{-1}\right)$	SDC	CR	RMSE $\left(cm^{-1}\right)$	SDC	CR
Simulated	1.2±0.6	0.58±0.27	0.8±0.13	1.31±0.59	0.71±0.22	0.9±0.09
Experimental 690 nm	0.95±0.054	0.59±0.06	0.88±0.04	2.56±0.11	0.66±0.05	0.96±0.03
Experimental 830 nm	0.70±0.029	0.49±0.03	0.69±0.03	1.98±0.08	0.63±0.03	0.94±0.02

**Fig. 9.**
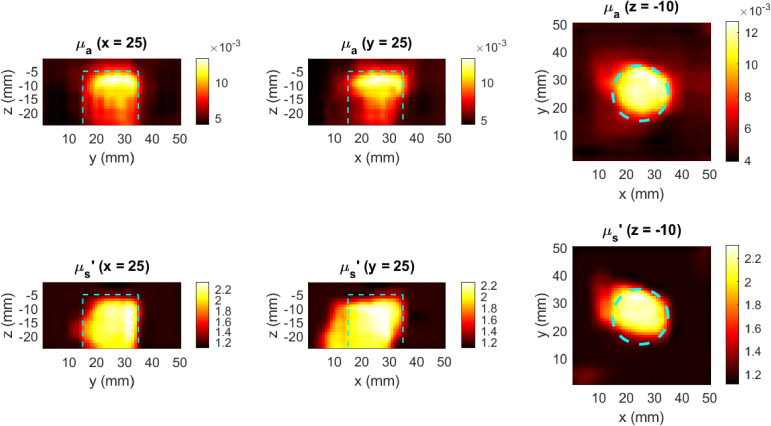
Recovered phantom optical properties from 200 unstructured measurements with a FD-DOT probe at 690 nm. The dashed cyan line indicates the true anomaly location.

**Fig. 10.**
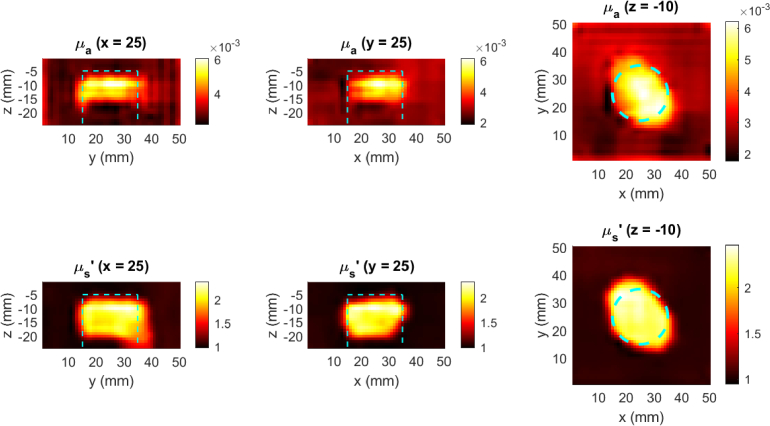
Recovered phantom optical properties from 200 unstructured measurements with a FD-DOT probe at 830 nm. The dashed cyan line indicates the true anomaly location.

Figures 9 and 10 show cross-sections of representative examples of the reconstructed phantom optical properties using 200 randomly sampled measurement positions from experimental scans at 690 and 830 nm respectively, using the model described in Section 2.3. The cyan dashed lines indicate the true outline of the tumor emulating anomalies, illustrating the high spatial accuracy of the reconstructed anomalies.

**Fig. 11.**
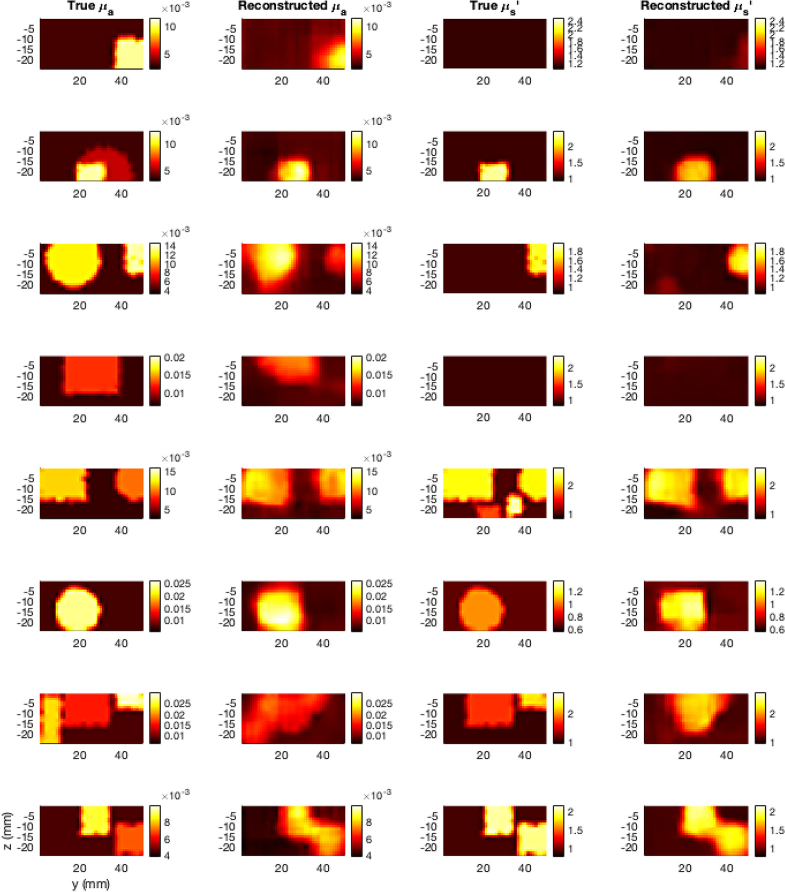
Cross-sections of eight randomly selected reconstructions of high-density simulated scans (one per row). Each reconstruction was produced using the same model based on a different random scanning pathway of 190-200 discrete probe positions on the tissue surface. The ground-truth and reconstructed $\mu_{a}$ and $\mu_{s}^{\prime }$ slices show the maximally perturbed x-axis cross-section for each example.

Figure 11 shows cross-sections of eight reconstructions of digital phantoms with different background and anomaly optical property distributions. These were chosen by selecting the first eight examples from the unseen test examples which used 190-200 measurements and contained tumor-emulating anomalies (i.e. the same criteria used to calculate the metrics in Table 3). Note that all of these were reconstructed using the same trained DL-DOT model but different scanning pathways.

**Fig. 12.**
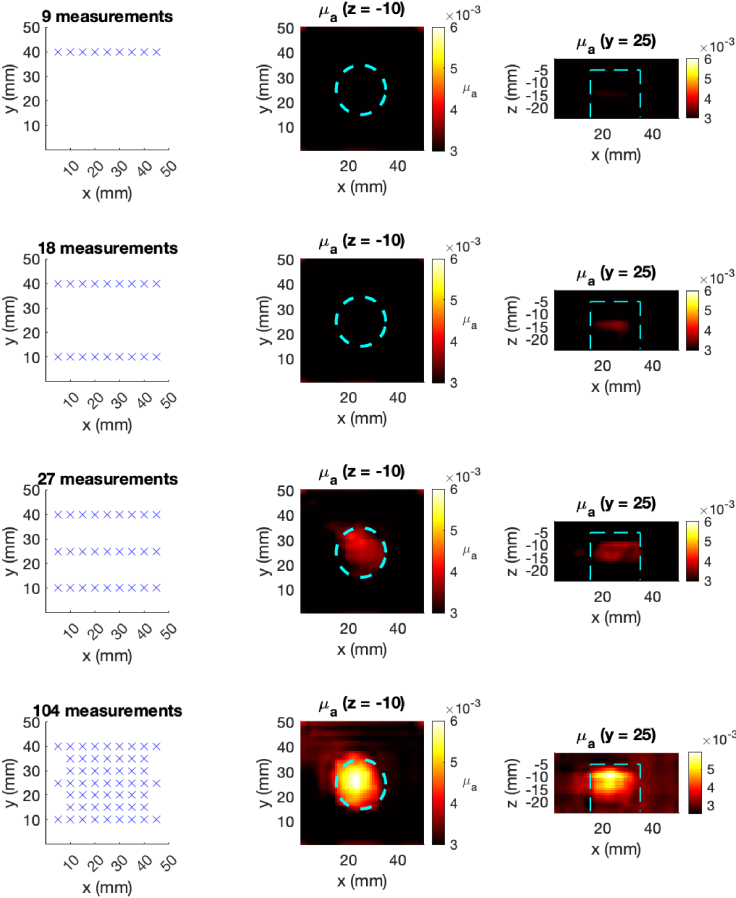
Example of image-guided scanning with a mechanical FD-DOT probe at 830 nm. The left column shows the incremental measurement positions on the phantom surface, and the middle and right columns show the corresponding recovered $\mu_{a}$ . The dashed cyan lines indicates the true anomaly location.

Figure 12 shows an example of image-guided scanning using the transformer encoder with experimental data. An image is progressively constructed using from incoming unstructured measurements, starting with a sparse line scan to detect possible features of the tissue, followed by higher density scan over an identified region of interest.

## 4. Discussion

The results demonstrate that the transformer architecture can effectively encode unstructured DOT measurements, capturing sufficient information to reconstruct informative optical property images. While it is not possible to perform a direct statistical comparison between the transformer+CNN model and previously published DL-DOT models, since they have been trained on different datasets and for different tasks (previous models cannot handle the unstructured scan data used here), the equivalent metrics reported for the multi-parameter FDU-Net model in [23] provide a general idea of relative reconstruction quality, since the simulated dataset used in that work was generated based on the same randomised parameters ( $\mu_{a}$ and $\mu_{s}^{\prime }$ distributions; anomaly numbers, sizes, shapes), and the model was tested on the same physical phantom (referred to as Phantom 2 in that study). In the current study, the average SDC scores for the high-density reconstructions are almost all over 0.5, representing majority overlap between the true and reconstructed anomalies. This is reflected in Figs. 9, 10, and 11 by qualitatively accurate anomaly edges. Furthermore, the SDC scores with ~144 measurements per scan (the number of measurements used in [23]) encompass within 1 standard deviation the scores reported in [23]. The one exception was the 830 nm $\mu_{a}$ reconstruction (Fig. 10), in which the significantly lower SDC score (0.49) corresponds to the lower half of the anomaly, which was never recovered regardless of the sampling pattern. Since the depth and lateral position of the reconstructed anomaly was generally accurate, and the 690 nm reconstruction captured greater depth (Fig. 9), it is possible that this was caused by imperfect coupling of the 830 nm source during the phantom scan. These SDC scores demonstrate that the model can extract spatial features such as anomaly depth, size and shape, by integrating information from heterogeneously related, multi-distance measurements, with comparable performance to a geometry-specific model.

Furthermore, excepting the same 830 nm $\mu_{a}$ image, the RMSE scores of the ~144 measurement examples (both experimental and simulated) are either lower on average, or encompass within one standard deviation, those reported in [23], suggesting similar overall accuracy as compared to the multi-parameter FDU-net.

The CR scores reported in this study, although considerably higher than those usually recovered with regularisation-based approaches, were consistently lower than those reported in [23]. This is thought to be because of the region-of-interest-weighted loss function used in that study (adapted from [22]), which was designed to preferentially penalize errors in anomaly regions over background regions.

The recovered $\mu_{a}$ and $\mu_{s}^{\prime }$ values for simulated examples with homogeneous optical properties were on average within 10% of the true values, showing that the transformer can infer absolute $\mu_{a}$ and $\mu_{s}^{\prime }$ values from realistically noisy measurements. It is noted that bulk estimation with real system measurements may not be as accurate, since the simulated scans assume perfect coupling of all optodes with the tissue surface simultaneously, which is difficult to achieve with a physical system, especially with a mobile probe. The RMSE scores were significantly lower in both the simulated and experimental examples containing tumor-emulating anomalies, which is expected due to a combination of the correspondingly lower signal-to-noise ratio, underestimated anomaly contrast, and background artifacts artifacts caused by imperfect anomaly localisation.

The similarity in SDC and CR metric scores between the simulated and experimental test examples, and the qualitative similarity between the corresponding example reconstructions (Figs. 9, 10, 11) demonstrates the efficacy of training DL-DOT models exclusively on simulated data, and indicate that the model is approximating a general solution to the DOT inverse problem, rather than over-fitting to specific characteristics in the simulated training data. The difference in the distribution of metrics scores between the experimental and simulated examples was greater for RMSE than for the spatial metrics, though the experimental scores were still generally lower or within one standard deviation of the simulated scores, This could be partially due to the specific optical property distributions of the phantoms, e.g. the scattering of the phantom in 690 nm fiber probe scan is almost 2 standard deviations from mean value in the training data, which could explain the significantly higher error in recovered $\mu_{s}^{\prime }$ , but could also be indicative of either imperfect measurement calibration or imperfect ground-truth charaterisation of the phantom. The higher variance in all metric scores for the simulated examples (calculated across many different simulated tissue examples) reflects the significant effect of tissue configurations on the quality of the reconstructed images. This is consistent with the physical limitations of DOT to accurately characterise certain tissues, due to factors such as tissue density [36] and anomaly depth [37] due to the signal-to-noise ratio in NIR measurements. On the other hand, the variance within each experimental example is due only to differences in scanning protocol, since these were calculated for multiple measurement samples from individual scans.

It is noted that the SDC and CR scores were consistently higher for the recovered $\mu_{s}^{\prime }$ as compared to $\mu_{a}$ . This has been previously observed in [38] and [23], and is thought to be due to the differences between phase and amplitude sensitivity, specifically that the phase sensitivity to scattering has a greater maximum depth and a steeper spatial gradient. This results in sharper edges and higher contrast in the $\mu_{s}^{\prime }$ reconstructions (visible in the $\mu_{s}^{\prime }$ images in Figs. 9, 10). Furthermore, while all reconstructions predict lower contrast for the deeper parts of the anomalies, due to the physical limits of sensitivity for a given SDS (1/2 SDS is often used as a rule of thumb for depth sensitivity, depending on optical properties), the bottom edge of the recovered anomalies is significantly deeper in the $\mu_{s}^{\prime }$ reconstructions. It may be possible to leverage the superior spatial characteristics of the $\mu_{s}^{\prime }$ reconstructions to improve the $\mu_{a}$ . While the 3D conv decoder used in this study allows sharing of spatial features between the two parameters, this could be enhanced either by expanding the loss function to include spatial similarity between parameters, or simply by reducing the proportion of training examples where $\mu_{a}$ and $\mu_{s}^{\prime }$ vary independently (in breast imaging strong $\mu_{a}$ and $\mu_{s}^{\prime }$ perturbations tend to be co-located due to shared underlying causes e.g. tumor growth).

The primary contribution of this work is to demonstrate the use and benefits of explicit spatial encoding in DL-DOT. As explained in Section 2.3 the transformer encoder calculates the relationships between measurements based on learned linear transformations of a set of continuous variables representing spatial position (in this case x, y, sin( θ ), and cos( θ ) of the probe), rather than relying on the positions or relative positions of measurements in the input space to infer relationships (i.e. implicit or innate spatial encoding) as in all previous DL-DOT models. Decoupling the relationship between discrete input positions and scan geometry in this way confers multiple practical benefits for developing DL-DOT for clinical use. Firstly, it makes the model more generalisable - a single trained transformer can be used for multiple scans with different scanning pathways, spatial precision, and different sized scan areas. This reduces the need to train new models for different use cases, which is important for clinical DOT because differences in individual morphology and tissue features may prevent replicating the scanning protocol used in the training data, and simulating subject-specific scan data is computationally expensive and time consuming. Secondly, it exploits the inherent spatial relationships in DOT data without enforcing structural assumptions. CNN encoders extract local features hierarchically, limiting spatial information transfer according to filter size and resolution, and FC encoders discard it all together. By contrast, the transformer’s attention mechanism allows it to attend to any part of the input based on both spatial and feature information, irrespective of its position in the input sequence. This enables the model to establish global features, like background optical properties and anomaly count, without being dependent on resolution or scan size [39]. Thirdly, it is more scalable for dealing with high-density DOT data. In FC or conv measurement encoders, the number of parameters scales quadratically with with the resolution of the input $O(r^{2})$ , whereas the transformer’s parameter count is independent of resolution O(1) , which allows the transformer to scale directly to arbitrarily precise/high density measurements without increasing the training costs of the model.

These features make the transformer encoder well suited to unstructured scanning, which together with the high-speed inference of neural networks and increasingly mobile hardware, enables DOT to be used for continuous, 3D image-guided scanning, as illustrated in Fig. 12. In this study, the average time taken to produce a single reconstruction was 0.07±0.01 sec. This is significantly slower than the FDUnet models reported in [22] and [23], which both took ~0.02 for reconstructions of a similar size and resolution, but is still significantly faster than gold standard analytical techniques (around 3-10 minutes), and translates to an effective imaging speed of 14 Hz, similar to modern ultrasound systems used for structural breast imaging. Interestingly, the gradients of all of the curves in Fig. 8 share a characteristic flattening beyond ~100 measurements, i.e. in scans where there were on average >4 measurements per $cm^{2}$ of the scan area, representing diminishing returns in the practical trade-off between scan density (and therefore measurement time) and image quality.

In addition to conferring benefits to the specific use case demonstrated here - i.e. unstructured scanning with a mechanical probe - the transformer encoder provides a scalable method for incorporating contextual information in DL-DOT. The ’context vector’ used here to embed the position and angle of the probe with the corresponding measurement data, could be extended to encode additional degrees of freedom, and thus enable additional degrees of generalisablity. For example, extending the positional information to 3D may enable the model to learn tissue-geometry independent and therefore subject-independent representations. Similarly, explicitly encoding modulation frequency may enable frequency-independent models, which are more suited to use across multiple imaging systems and measurement types (e.g. CW and FD). Increasing generalisability through explicit context encoding is a promising direction for future research in DL-DOT and its potential as a clinical tool, but will also incur new challenges, primarily the complexity and computational cost of designing, simulating and storing sufficiently numerous and realistic training data that capture the full range of variation across all contextual variables, to enable the model to effectively learn and generalise these relationships.

The model described and evaluated in this work constitutes a proof of concept for the DOT transformer encoder, however there are several important limitations of the transformer+CNN architecture as described here. Indeed there is significant work to do before applying this model *in vivo*. Firstly, the data used in this study represent a simplified case of DOT for human breast imaging, which models the most relevant tissue characteristics for breast cancer monitoring - i.e. optical property distributions, and the contrast and spatial differentiation between background tissue and tumor-like anomalies. As such, the network evaluated in Section 2.5 is not expected to generalise to *in vivo* data from the current training data, and the results of this study do not therefore constitute proof of clinical feasibility. In order to generalise effectively to *in vivo* data, other characteristics of human tissue which affect DOT measurements - e.g. skin pigmentation and heterogeneous background tissue types - should also be modeled, and randomised within the training data based on biologically realistic distributions. This has been demonstrated in previous DL-DOT analysis studies with the use of more realistic digital breast models [40], and will be prioritised in future work. It is possible that inhomogeneous background optical properties could make it slightly more challenging to recover accurate images, however since the inhomogeneities in healthy breast tissue are significantly smaller than the differences between healthy and tumor tissue, this is not expected to have a dramatic impact on the recovered tumor characteristics. Indeed, in [22], a DL-DOT model trained on simplified tissue models similar to ours generalised well to clinical breast data, enabling accurate localisation of a tumor identified in a co-registered X-Ray. It should also be noted that the benefits of explicit spatial encoding for integrating spatial information are expected to persist regardless of the complexity of the tissue configuration.

Secondly, the conv decoder is limited (in the same ways as conv encoders) to a predetermined size and resolution voxel representation, and so is not well suited to represent realistic variation in tissue shape and size. Also, as discussed in the context of measurement integration, the CNN’s local spatial filtering, while efficient for inferring spatially-recurring features, limits the network ability to model complex long-range dependencies which may be present in *in vivo* data. While this work specifically focused on explicit spatial encoding for DOT measurements (i.e. the front end of the DL-DOT network), a variation of the same architecture may provide similar benefits in the decoding task. Thirdly, and specifically with respect to the proposed ’image-guided scanning’ use-case, the current network output does not discriminate densely measured areas, where the reconstruction is likely to be accurate, from sparsely measured or unmeasured areas, where it is likely to be inaccurate. This could be addressed in future models, e.g. by visually augmenting the output with the estimated sensitivity map based on the input measurement positions, or by extending the loss function to enable probabilistic output values for each voxel, as in variational-autoencoder [41].

## 5. Conclusion

This work demonstrated the use and benefits of a transformer encoder neural network architecture for extracting clinically relevant features from unstructured DOT scan data. A single trained instance of the model can integrate arbitrary spatial measurement sequences, removing the need to follow a prescribed measurement protocol - demonstrated here with experimental scan data from a two-wavelength FD-DOT system and a breast tumor-emulating optical phantom. Evaluation of the recovered DOT images based on experimental data indicates accurate absolute optical property values and spatial characteristics, and are supported by a large sample of simulated examples. This technique affords greater flexibility and scalability as compared to previously proposed models, and compliments the practical benefits of modern handheld FD-DOT systems for real-time image-guided scanning.

## Data Availability

The data presented in this article are publicly available at [42].
